# Progress in Obstetrics

**Published:** 1901-03-09

**Authors:** 


### Progress in Obstetrics.

(*Continued from page* 387.)

**Puerperal Sepsis.**—Dr. Ranken Lyle[13] defines puerperal sepsis as nothing more or less than a surgical fever arising from the infection of a wound, the only difference being (1) the large extent of surface liable to infection; (2) the structures involved; (3) the presence of a quantity of dead material which easily lends itself to infection; and (4) the fact that the patient is undergoing certain physiological changes, this perhaps rendering her more liable to infection. He discusses the different varieties of puerperal fever under the following headings: (1) sapræmia or septic intoxication due to a local cause, (2) septicæmia or acute septic infection, and (3) pyæmia. With regard to the treatment of sapræmia he recommends vaginal and intrauterine douching with creolin solution; and when there is reason to believe that some of the membranes or part of the placenta have been retained, gentle curetting with Rheinstadter's flushing curette. In septicæmia besides local treatment, such as douching and plugging the uterus with iodoform gauze, he recommends large doses of alcohol, with strychnine and digitalis. He is not in favour of using antistreptococcic serum in these cases, and considers that the cases of puerperal septicæmia reported as successfully treated with the serum have probably been cases of sapræmia which were wrongly diagnosed as septicæmia. He says that by injecting a patient with a quantity of this serum it is quite possible to produce a condition known as antistreptococcic neuritis, several cases of which have been observed both in this country and on the Continent. Dr. Pryor,[14] writing on puerperal septicæmia, also condemns the use of the antistreptococcic serum, and thinks that in the thrombo-phlebitic form of septicæmia abdominal hysterectomy preceded by intravenous transfusion of saline solution gives the patient the best chance; in cases of lymphatic septicæmia he recommends curettage of the uterus followed by irrigation with saline solution and packing the uterus with iodoform gauze; in addition to this he makes a broad incision into the posterior cul-de-sac, evacuates the serum or pus which is often present in the pelvis, and then packs the pelvis with iodoform gauze.

**Cæsarian Section.**—W. J. Sinclair[15] records ten successful cases of Cæsarian section, and in his general remarks on the cases says that the selection of the time for operation is a point on which a good deal of difference of opinion appears to exist. The operation used to be looked upon as a last resort and a dire necessity; now it is usually done without delay, while the condition of the patient and the parts to be operated on are comparatively favourable. He thinks the most favourable time should be chosen, regardless as to whether labour has commenced or not. He recommends that the incision in the uterus should not be made until that organ has been drawn forward out of the abdominal wound, and that an elastic tube should always be put round so as to be ready to compress the uterine vessels in case of emergency. As to the length of the uterine incision he thinks that one of 4½ inches is usually required. With regard to the position at which the incision into the uterus ought to be made, he does not favour the transverse one at the fundus, but thinks the middle third in front and in the median line the best. With increasing experience and confidence he thinks it advantageous to abandon drainage. Dr. David St. John[16] also thinks it advisable to bring the uterus out of the abdomen before incising it. He believes that excessive uterine hæmorrhage can always be effectually controlled by an assistant grasping the uterus firmly low down near the neck with both hands, and he recommends that the cervix should be dilated before opening the abdomen.

[13] Lancet, Sept. 29th 1900, Dr. R. P. Ranken Lyle. [14] Med. Rec., Oct. 27th, 1900, Dr. W. R. Pryor. [15] Lancet, Jan. 19th, 1901, W. J. Sinclair. [16] Med. Rec., August 1900, Dr. David St. John.

